# A New Strategy in Modulating the Protease-Activated Receptor 2 (Par2) in Autoimmune Diseases

**DOI:** 10.3390/ijms26010410

**Published:** 2025-01-06

**Authors:** Lynn Khoon, Ron Piran

**Affiliations:** The Azrieli Faculty of Medicine, Bar-Ilan University, Safed 1311502, Israel; lynn.khoon@live.biu.ac.il

**Keywords:** autoimmunity, inflammation, regeneration, regenerative medicine, G protein-coupled receptors (GPCR), protease-activated receptors

## Abstract

Autoimmune diseases are complex conditions characterized by immune-mediated tissue damage and chronic inflammation. Protease-activated receptor 2 (Par2) has been implicated in these diseases, exhibiting dual roles that complicate its therapeutic potential. This review examines the perplexing functions of Par2, which promotes inflammation through immune cell activation while facilitating tissue healing in damaged organs. By analyzing findings across diverse autoimmune conditions, including rheumatoid arthritis, type 1 diabetes, and inflammatory bowel disease, we highlight how the context and location of Par2 activation determine its effects. Recent studies from our laboratory have resolved some of these contradictions by distinguishing Par2’s immune-mediated inflammatory roles from its tissue-reparative functions. These insights pave the way for context-specific therapeutic strategies, such as selective Par2 modulators, that can mitigate inflammation while enhancing tissue repair. However, achieving such precision in modulation remains a significant challenge, necessitating further research into Par2’s signaling pathways. This review underscores Par2’s complexity and its transformative potential in autoimmune disease management, offering a nuanced perspective on its duality and therapeutic implications.

## 1. Introduction

The prevalence of autoimmune diseases is rising drastically worldwide, driven by environmental and lifestyle changes such as climate shifts, air pollution, infections, stress, and individual lifestyle choices. Despite the growing awareness and recognition of autoimmunity, the precise causes remain elusive [1].

The immune system can be divided into two primary components: innate immunity and adaptive immunity. While these components operate through different mechanisms, they work together to offer protection from potentially harmful elements [2]. When a pathogen is identified, innate immunity is activated first, followed by adaptive immunity, which can contribute to the development and persistence of autoimmune diseases [3]. This activation sets off complex cascades involving molecules, cells, and organs that work in concert. Certain factors can sometimes disrupt these immune processes, leading to malfunctions that may result in the production of self-targeting autoantibodies. These autoantibodies can damage our tissues and give rise to autoimmunity [4].

Autoimmunity is a general term that encompasses various autoimmune diseases. Although these diseases share certain characteristics, they differ significantly in complexity and presentation, with each condition exhibiting its own unique set of challenges [5].

**Figure 1:**
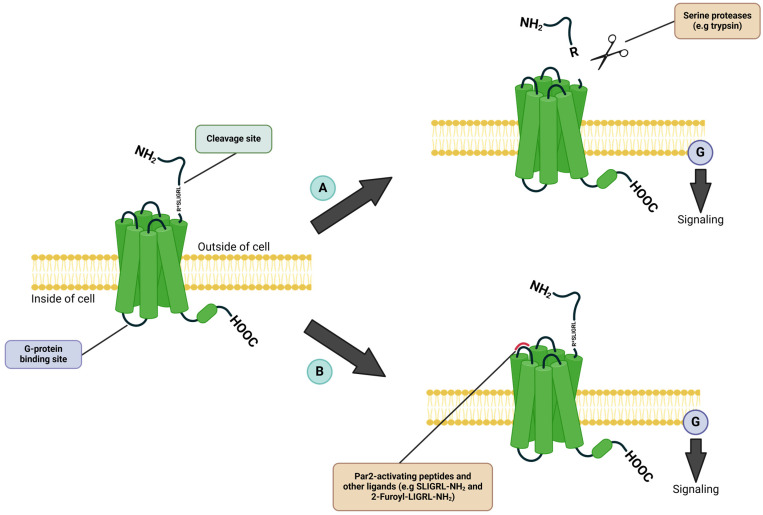
**Par2 activation mechanisms.** (**A**) Protease-activated receptor 2 (Par2) is activated by serine proteases (e.g., trypsin) that cleave it at its N-terminus at a specific site. Subsequently, a tethered ligand that binds to the second extracellular loop is exposed, resulting in Par2’s activation. (**B**) Par2 can be activated by synthetic agonists administered pharmacologically, which are known as Par2-activating peptides, that mimic the tethered ligand and bind within the second extracellular loop to activate the receptor (e.g., SLIGRL-NH_2_(Indicated by *), 2-Furoyl-LIGRL-NH_2_).

Protease-activated receptor 2 (Par2) is a membrane-bound G protein-coupled receptor (GPCR) with seven transmembrane domains that is activated by serine proteinases, such as trypsin [6], or by synthetic peptides (Figure 1). The activation by trypsin occurs through the proteolytic cleavage of the N-terminus region of the receptor, resulting in the exposure of a tethered ligand that undergoes a conformational change [7]. Subsequently, this ligand binds to Par2 in an intramolecular manner, causing it to activate and initiate intracellular signaling pathways within the cell with the help of different G protein α subunits, including Gαq/11, Gαi, Gα12/13, Gαi/oGαq/11, and Gαi/o [8]. In addition to G protein-dependent pathways, Par2 can trigger G protein-independent signaling by recruiting β-arrestin to its carboxy-terminus. Upon Par2’s activation by trypsin, it undergoes receptor phosphorylation, which regulates its localization on the cell surface. Both constitutive activation (by proteases) and activation by agonists cause receptor internalization, followed by degradation to terminate the signal [8,9]. Once Par2 is activated, it can initiate various downstream effects, including calcium influx via Gαq, increased cAMP levels through Gαs, elevated Rho-Kinase activity mediated by Gα12/13 [10], increased Rho-Kinase activity by Gα12/13 [7], recruitment of β-arrestin-1 and β-arrestin-2, and ERK1/2 phosphorylation [11], ultimately leading up to Par2 internalization and degradation [8].

Par2 modulators have been developed over the years, including both agonists and antagonists that enable precise modulation of Par2 signaling. Notable agonists include SLIGRL-NH_2_ [12,13] and 2-Furoyl-LIGRL-NH_2_ [14,15], which are synthetic peptides designed to mimic the tethered ligand-binding properties of Par2, thereby initiating its activation. Compounds like FSLLRY-NH_2_ [16,17] and GB88 [18] have emerged as effective antagonists, acting as inhibitors of Par2-activated Ca^2+^ release, which can be induced either by native trypsin or by synthetic agonists [17,18].

Par2 is known to participate in various processes, including pain [19,20,21], skin irritation and itching [22,23], chronic arthritis [24,25], and inflammatory bowel diseases [26,27,28]. Furthermore, studies have identified its fundamental role in numerous inflammatory processes in the body. It has been shown to exacerbate hepatic damage [29], type 1 diabetes [30], and neuron injury and neurotoxicity [31]. Contrarily, it has also been shown to play a role in regeneration. It enhances epithelial wound healing in inflammatory bowel diseases [32], myelin development and repair [33], and hepatic and pancreatic regeneration [29,30,34].

The contradictory roles of Par2 can pose challenges for further translational approaches, as in some cases it can exacerbate inflammation and in other cases it can promote healing. Inflammation and regeneration are two processes that usually go together, as inflammation is a crucial step in recovery [30]. While this can be beneficial, it can also be destructive in autoimmune conditions, thus aggravating inflammation while obstructing healing. We recently addressed the duality of Par2 in inflammation and regeneration and demonstrated that the role of Par2 depends on the initial system in which it is activated. We showed that its activation in the immune system intensified inflammation, whereas its activation in damaged tissue promoted regeneration [8,29,30].

## 2. Par2 in Autoimmune Diseases

Autoimmune diseases represent a diverse group of conditions characterized by the immune system’s failure to distinguish self from non-self, resulting in tissue damage and chronic inflammation. These diseases often involve a complex interplay between genetic predispositions, environmental triggers, and immune dysregulation. Par2 has emerged as a significant player in autoimmune pathophysiology, displaying dual roles in experimental models. Par2 activation exacerbates inflammation in immune cells while promoting tissue repair in damaged tissues. This context-dependent duality, clarified by recent studies from our lab, highlights the receptor’s distinct roles in autoimmune diseases [8,29,30]. In the following sections, we explore Par2’s involvement across various autoimmune conditions, highlighting its complex roles and the implications for therapeutic interventions.

### 2.1. Autoimmune Hepatitis

Autoimmune hepatitis is an immunoinflammatory chronic disease of the liver [35]. The development of the disease encompasses multiple factors and interactions between environmental triggers, liver tolerance, and impaired immunological processes [36]. The disease onset can be elicited by autoreactive CD4 and CD8 T cells, which are recruited in response to self-tolerance breaks by environmental triggers [35]. Diagnosis can be challenging since the disease can branch out into different subtypes and can vary in its clinical manifestations, as it can be a very dynamic disease, making it difficult to diagnose. It is currently recommended to take a liver biopsy as the first step in diagnosis, which can provide initial information regarding the severity of the disease [35,36].

**Figure 2:**
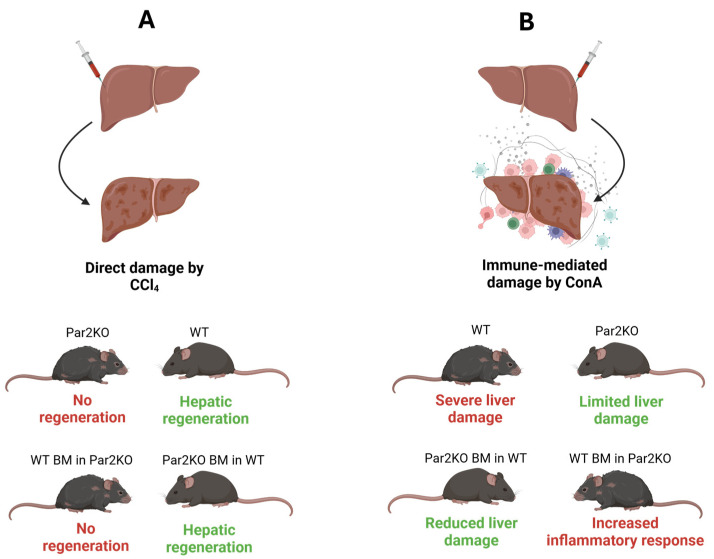
**Par2’s role in autoimmune hepatitis.** The effects of Par2 signaling on liver regeneration and inflammation in wild-type (WT) and Par2 knockout (Par2KO) mice in two different models of liver injury. (**A**) In the chemically induced liver damage model using CCl_4_, WT mice exhibited significant hepatic regeneration, whereas Par2KO mice showed limited signs of regeneration. The bone marrow transplantation experiment showed no significant difference in liver regeneration between transplanted and non-transplanted mice. (**B**) In the immune-mediated liver damage model using ConA, non-transplanted WT mice exhibited severe liver damage, whereas non-transplanted Par2KO mice showed limited liver damage. Among the transplanted groups, Par2KO mice transplanted with WT bone marrow exhibited an increased inflammatory response, while the reciprocal experimental group showed reduced liver damage.

We demonstrated that Par2 exhibits dual roles in autoimmune hepatitis, with its effects depending on the site of activation [8]. Using immune-mediated (ConA) and direct damage (carbon tetrachloride, CCl_4_) hepatic injury models, we showed that Par2KO mice had greater hepatocellular damage in the direct damage model (CCl_4_) but were protected from inflammation in the immune-mediated hepatitis model (ConA) (Figure 2A,B). Further experiments with bone marrow transplantation revealed that hematopoietic Par2 was required for ConA-induced inflammation, while hepatic Par2 was essential for regeneration in the CCl_4_ model. These findings underscore the critical role of Par2’s activation site in determining its function as either a driver of inflammation or a promoter of tissue repair [29].

### 2.2. Type 1 Diabetes

Type 1 diabetes (T1D) is an autoimmune disorder where macrophages and CD4+ and CD8+ T cells infiltrate the islets of Langerhans, destroying insulin-producing β-cells. This progressive β-cell loss ultimately results in severe insulin deficiency, leading to hyperglycemia in affected individuals [37]. Despite extensive research efforts and significant advancements in our understanding of T1D over the past two decades, the precise etiology of this chronic autoimmune condition remains elusive. Although it may initially appear straightforward due to its extensive study, it is increasingly recognized as a multifaceted interplay of various factors that can vary considerably among individuals [38].

**Figure 3:**
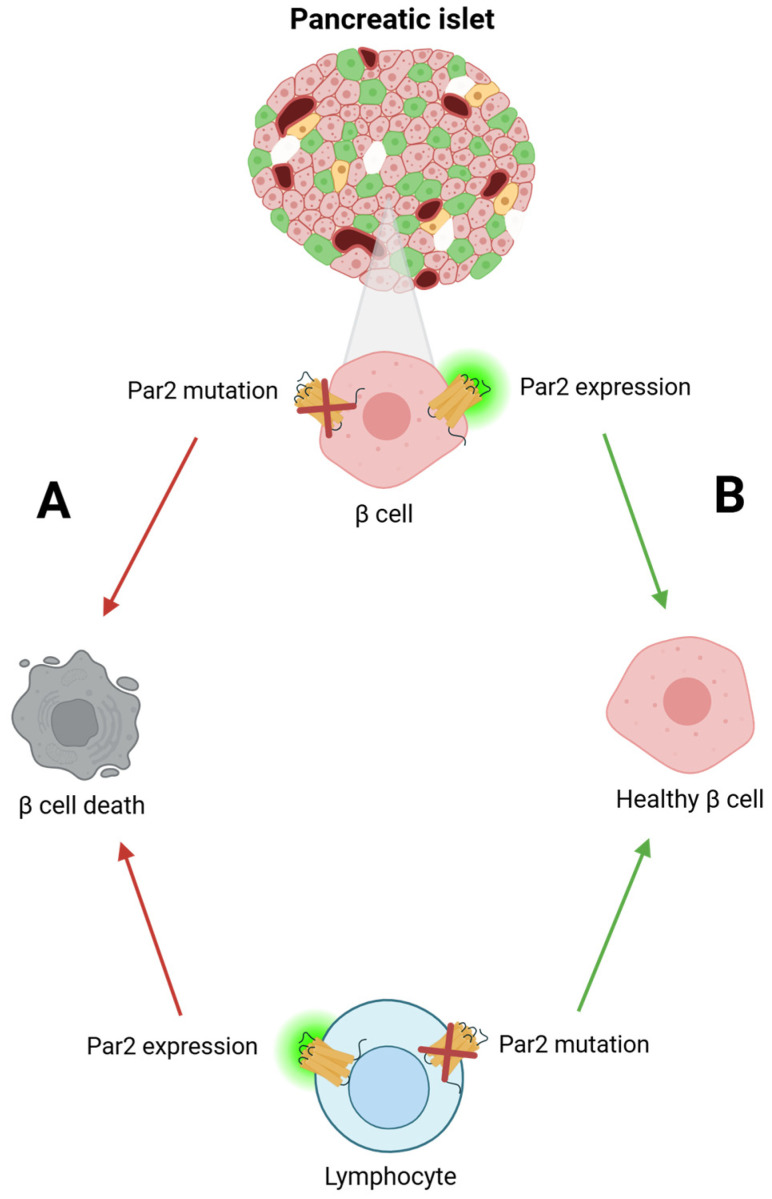
**Par2’s role in type 1 diabetes (T1D) pathogenesis.** ((**A**), red arrows) In the absence of Par2 in β-cells, these cells are not protected from autoimmune attack and are susceptible to destruction by Par2-positive lymphocytes, ultimately resulting in the development of diabetes. ((**B**), green arrows) When Par2 is present in β-cells, it confers protection against autoimmune attacks. Conversely, Par2 expression in lymphocytes promotes autoimmunity (red arrow, lower panel). Notably, in mice with Par2-deficient lymphocytes (green arrow, lower panel), β-cells are fully protected from immune-mediated destruction, and diabetes does not develop.

In T1D, the primary problem associated with this progressive disease is the continuous destruction of β-cells, which leads to elevated blood glucose levels, which can contribute to the development of additional health complications if left untreated. One possible approach to address this issue involves the inhibition of the destruction of β-cells through the activation of Par2. Par2 is well-known for its reparative properties in various tissues [29,30,32,33,34]. Our recent findings highlight the critical role of Par2 in safeguarding β-cells from damage in type 1 diabetes. Specifically, when Par2 was genetically deleted in β-cells, we observed significant damage to the islets of Langerhans, as demonstrated by the loss of β-cell integrity and increased susceptibility to autoimmune attack (Figure 3A). Conversely, Par2 deficiency in lymphocytes conferred protection against β-cell destruction, effectively preventing the onset of diabetes (Figure 3B). These contrasting outcomes underscore the dual role of Par2 in mediating tissue protection and immune regulation [30]. Additionally, when Par2 was knocked out in the retina that served as a control, we observed a distinct increase in Par2 expression within the β-cells that survived the infiltration. This indicates that Par2 plays a critical role in the response of β-cells to damage, with the upregulation of the receptor serving as a mechanism for pancreatic tissue protection.

This led us to conclude that the contradicting roles of Par2, where sometimes it aggravates inflammation and other times it promotes regeneration, depend on its initial activation site. As seen in the pancreas, when Par2 was activated in the tissue, it granted protection against the destruction of β-cells, whereas its activation in the immune system exacerbated the inflammation surrounding the islets. These findings may be the key to developing therapeutic strategies in attempts to tackle autoimmune conditions [30].

### 2.3. Rheumatoid Arthritis

Rheumatoid arthritis (RA) is a multifactorial autoimmune disease marked by chronic joint inflammation, which can lead to bone and cartilage damage or even disability [39]. Although the exact mechanisms are not fully understood, rheumatoid factor (RF) and anti-citrullinated protein antibodies (ACPA) are known to contribute to disease progression and significantly impact susceptibility to RA [40]. One of the strongest genetic risk factors for RA is the *human leukocyte antigen (HLA)* gene. Within the *HLA class II* region, the *HLA-DRB1* gene contains a conserved amino acid sequence that is shared across multiple risk alleles associated with RA. Notably, the *HLA* locus has been predominantly linked to seropositive RA and elevated levels of antibodies targeting citrullinated proteins [40].

The most noteworthy immune processes are the ones that take place in the joint synovium and synovial fluid. During those processes, synovial macrophages release cytokines such as tumor necrosis factor α (TNF-α), interleukin-1 (IL-1), and interleukin-6 (IL-6), which co-stimulate the activity of osteoclasts, along with inflammation and fibroblast-like synoviocytes (FLS), ultimately leading to bone erosion progression [41]. Additionally, activated FLS can produce matrix metalloproteinase (MMP), leading to cartilage degeneration. Evidence suggests that Par2 plays a role in the pathology of RA including synovial hyperplasia, cartilage destruction, osteophyte formation, and pain [42,43].

**Figure 4:**
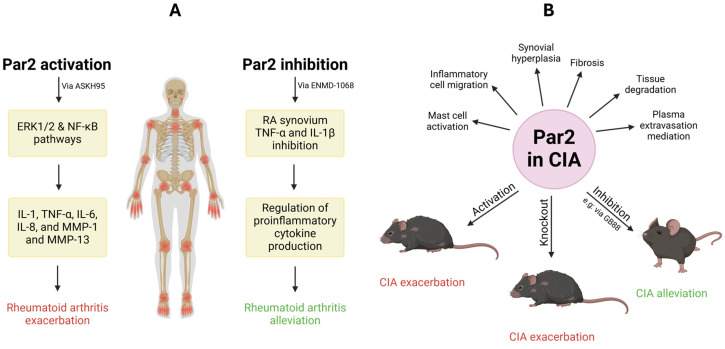
**Role of Par2 in rheumatoid arthritis and collagen-induced arthritis.** (**A**) The agonist ASKH95-induced activation of Par2 triggers the ERK1/2 and NF-κB signaling pathways that consequently induce pro-inflammatory cytokines and mediators including IL-1, TNF-α, IL-6, IL-8, MMP-1, and MMP-13. This cascade leads to the exacerbation of rheumatoid arthritis. On the other hand, the inhibition of Par2 using the antagonist ENMD-1068 reduces TNF-α and IL-1β release from RA synovium, hence controlling pro-inflammatory cytokine production and eventually attenuating symptoms of RA. (**B**) In the collagen-induced arthritis model, mice with activated or mutated Par2 showed signs of CIA exacerbation, whereas inhibition of Par2 via the antagonist GB88 promoted CIA alleviation.

Par2 has been shown to contribute to RA inflammation. Upon activation, Par2 signaling through the ERK1/2 and NF-κB pathways promotes the production of pro-inflammatory cytokines and mediators, including IL-1, TNF-α, IL-6, IL-8, MMP-1, and MMP-13, thus exacerbating disease progression (Figure 4A). The same study showed that levels of PRO-Par2, a peptide released following Par2 cleavage and activation, were elevated in RA patients compared to healthy controls, serving as a marker of Par2 activation [24].

Ferrell et al. showed the pivotal role of Par2 in mediating chronic inflammation through a combination of physiological, pharmacological, and genetic methods [25]. Using an adjuvant monoarthritis model of chronic inflammation, joint swelling in Par2KO mice was significantly inhibited compared to WT mice, exhibiting almost no histological evidence of joint damage. An intermediate phenotype was noted in mice that were heterozygous for Par2 gene disruption. Moreover, there was a substantial up-regulation of Par2 expression 2 weeks following the induction of inflammation, both in synovium and in other periarticular tissues. The use of Par2 agonists, in this case ASKH95, resulted in potent proinflammatory effects, inducing prolonged joint swelling and synovial hyperemia (Figure 4A). These findings highlight the key role of Par2 in mediating chronic inflammation, potentially serving as a therapeutic target for chronic inflammatory disease management [25].

Kelso et al. sought to investigate the hypothesis that Par2 plays a key role in the pathogenesis of rheumatoid arthritis, examining synovial Par2 expression and determining the effect of a Par2 antagonist on synovial cytokine production [44]. The authors discovered that Par2 was significantly up-regulated in RA synovium compared to controls. When using the Par2 antagonist ENMD-1068, the spontaneous release of TNF-α and IL-1β from RA synovium was remarkably inhibited in a dose-dependent manner. This study identified Par2 as a regulator of proinflammatory cytokine production in RA (Figure 4A) [44].

Collagen-induced arthritis (CIA) is the gold standard animal model for human rheumatoid arthritis (RA), as they share common pathological features including mononuclear cell infiltration, cartilage degradation, and synovial hyperplasia [45]. Despite these similarities, CIA differs from RA in that it lacks rheumatoid factor. Moreover, CIA is known to be a monophasic disease, and it affects both male and female mice similarly [46,47]. CIA is induced in genetically susceptible mouse strains through immunization with type II collagen (CII) emulsified in complete Freund’s adjuvant (CFA). Par2 is present in the bone, synovial lining, and cartilage of articular joints. It may contribute to inflammation by promoting protease-dependent tissue degradation, synovial hyperplasia, and fibrosis [48,49,50,51]. Furthermore, Par2 has been shown to mediate plasma extravasation, inflammatory cell migration, and mast cell activation, processes associated with arthritis and related chronic inflammatory diseases [52].

GB88, a Par2 antagonist with promising anti-inflammatory effects, was shown to alleviate CIA in rats (Figure 4B) [52]. Collagen injections led to progressive paw swelling in rats, particularly in the hind limbs. By day 20, all rats in the untreated CIA group developed arthritis, with an average swelling increase of 66% from baseline, reaching 90% by day 28. In contrast, GB88 treatment reduced the severity of arthritis; only 60% of the GB88-treated rats exhibited arthritis by day 28, with a maximum swelling of 25%, significantly less than the control group. GB88-treated rats showed lower inflammation scores (DAI) compared to untreated rats, who developed steadily worsening symptoms by day 28. Sham-treated rats showed no signs of arthritis [52].

Interestingly, although GB88, a Par2 antagonist, reduced arthritis severity in treated rats [52], Par2KO mice developed more severe arthritis compared to WT mice [51]. During the first 14 days of arthritis induction, Par2KO mice initially showed lower disease incidence and severity, but this was followed by a rapid and aggressive progression (Figure 4B) [51].

Crilly et al., who used Par2KO mice, obtained different results, showing less severe arthritis in Par2KO mice compared to WT mice. Histological analysis of the paws revealed cartilage erosion and inflammatory cell infiltration in WT mice, whereas Par2KO mice exhibited minimal damage, resembling healthy, untreated mice [53].

While Xue et al. [51] reported more severe arthritis in Par2KO mice compared to Crilly et al.’s findings [53], it is notable that Crilly et al. observed only up to 7 days post-arthritis induction, whereas Xue et al. extended their observations beyond 7 days, showing arthritis development in Par2KO mice after 14 days.

### 2.4. Psoriatic Arthritis

Psoriatic arthritis (PsA) is an inflammatory, immune-mediated musculoskeletal disease that can affect various organ systems, including the axial and peripheral joints, skin, entheses, and nails [54,55]. This chronic inflammatory condition, which manifests heterogeneously across tissues and clinical domains, can lead to joint destruction and disability if inflammation persists [56].

A study examined the role of Par2 signaling in inflammatory cell populations in synovial fluid (SF) and aimed to identify active serine proteinases that may function as Par2 activators. Their findings indicated that Par2 expression and signaling in monocytes/macrophages, which were the predominant cell types in PsA SF, had a substantial impact on the production of monocyte chemoattractant protein-1 (MCP-1). Additionally, tryptase-6 was identified as an active serine proteinase in SF that could trigger calcium signaling, partially via Par2 [57]. Findings indicated that Par2 is expressed by three primary monocyte/macrophage sub-populations in PsA SF [57,58].

**Figure 5:**
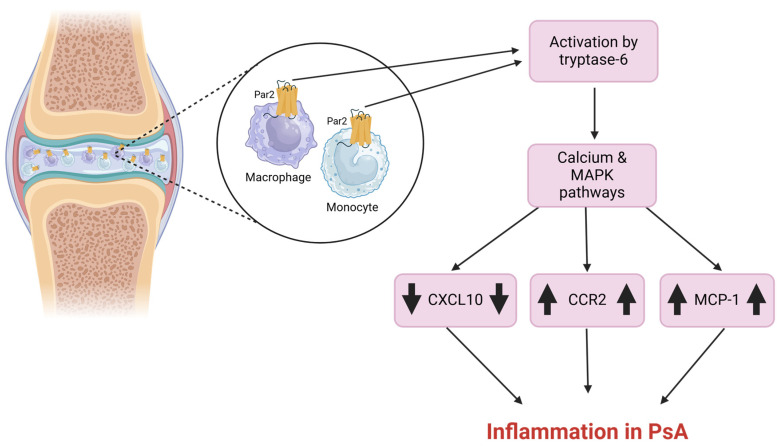
**Role of Par2 in psoriatic arthritis.** Par2’s activation and signaling in immune cells within the SF of PsA patients. Par2, expressed on monocytes and macrophages in PsA SF, is activated by tryptase-6, leading to the initiation of calcium and MAPK signaling pathways. This activation results in increased levels of CCR2 and MCP-1 and a concomitant decrease in CXCL10 expression in monocytes/macrophages, ultimately aggravating the inflammation in PsA.

Following Par2 activation, elevated MCP-1 levels were observed in monocytes/macrophages derived from PsA patients, along with an increase in CCR2-expressing monocytes/macrophages and a decrease in CXCL10 in SF (Figure 5). This suggests a potential mechanism by which Par2 may recruit monocytes/macrophages to the PsA joint [57]. Moreover, these specific immune cell populations were shown to respond to Par2 activation through calcium and MAPKinase signaling pathways. Tryptase-6 was used to activate these pathways, mimicking the effects of the Par2 agonist, 2fLI. This activation was confirmed in HEK-293 cells, which are known to respond to Par2 stimulation by this agonist [59,60]. The overall findings imply Par2’s key role in mediating inflammation in PsA.

### 2.5. Sjögren’s Disease

**Figure 6:**
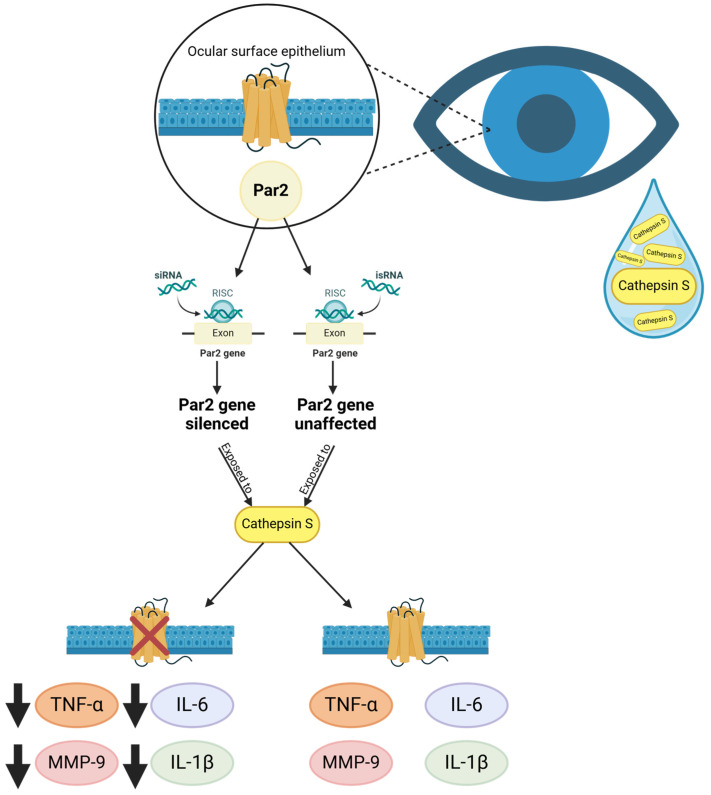
**Role of Par2 in Sjögren’s disease.** Human corneal epithelial cells were transfected with Par2 siRNA and isRNA (scrambled control) to investigate the role of Par2 in the inflammatory response induced by cathepsin S (CTSS). Cells were subsequently exposed to CTSS for 4 h. In cells transfected with Par2 siRNA, there was a significant reduction in the secretion of pro-inflammatory cytokines, including IL-6, TNF-α, IL-1β, and MMP-9, compared to the scrambled control. This reduction was observed both in gene expression and protein levels. The scrambled siRNA-transfected cells showed no reduction in gene or protein expression, indicating the specific role of Par2 in mediating the inflammatory response.

Sjögren’s disease is an autoimmune disease attributed to a wide variety of systemic manifestations, including lymphocytic infiltration, and loss of function of the lacrimal gland and salivary gland, leading to symptoms of dry eye and mouth [61]. Sjögren’s disease can exist independently as Sjögren’s disease or as part of other inflammatory conditions known as associated Sjögren’s disease. While dryness is the main symptom, most Sjögren’s disease patients also experience systemic symptoms such as fatigue and migrating muscular and joint pain [62] (Figure 6).

In Sjögren’s disease patients, levels of pro-inflammatory cytokines can change during or even before an immune cell infiltration in the lacrimal and salivary glands of Sjögren’s disease patients and mouse models. These cytokines are also found in tears and conjunctival epithelium [63], suggesting they could affect eye health. Cathepsin S (CTSS), a cysteine endopeptidase, is found in the tears of Sjögren’s disease patients and exhibits high activity in this environment. Klinngam et al. [63] showed that this specific enzyme can aggravate inflammation by stimulating pro-inflammatory gene and protein expression in a human corneal epithelial cell line, at activity levels found in Sjögren’s disease patient tears. Therefore, there is a possibility that these tear and ocular surface tissue cytokines might not necessarily derive from the lacrimal glands in Sjögren’s disease but may be induced via CTSS-mediated processes in ocular surface epithelia. Furthermore, their findings suggest that Par2 could play a role in mediating the acute inflammatory responses of the ocular surface induced by CTSS. These findings were based on an experiment performed on human corneal epithelial cells (HCE-T cells), which were transfected with Par2 siRNA, silencing the Par2 gene, along with scrambled siRNA that did not affect the gene. Following the cell culture exposure to CTSS, the cells with Par2 siRNA showed a significant reduction in the secretion of IL-6, TNF-α, IL-1β, and MMP-9 into the cell culture medium (Figure 6). Correspondingly, there was a decrease in the gene expression of IL-6 and TNF-α and the protein levels of IL-6 and MMP-9 within the cell lysates, whereas scrambled siRNA transfected cells showed no reduction in gene expression [63]. Interestingly, this process occurred before the CTSS-induced increase in Par2 protein expression at 24 h. This suggests that Par2 was already present on the cell surface or was recruited and was responsible for mediating the inflammatory response.

### 2.6. Systemic Lupus Erythematosus

Systemic lupus erythematosus (SLE) is an autoimmune disease characterized by impaired apoptotic clearance, heightened innate and adaptive immune activity, complement activation, immune complexes, and tissue inflammation leading to a self-sustained autoimmune process [64]. One of the most frequent phenotypes in SLE patients is lupus nephritis (LN), which may commence due to dendritic cell dysregulation and the production of autoantibodies by autoreactive B cells [65]. Certain factors such as CD4+ T cell imbalance, increased levels of pro-inflammatory Th17 and follicular helper T (TFH) cells, and a decrease in regulatory T (Treg) cells may all contribute to the occurrence and relapse of LN. Additionally, elevated levels of pro-inflammatory cytokines, which are involved in the progression of LN via specific signaling pathways (ERK/MAPK and NF-κB), can aggravate the severity of the condition [66].

**Figure 7:**
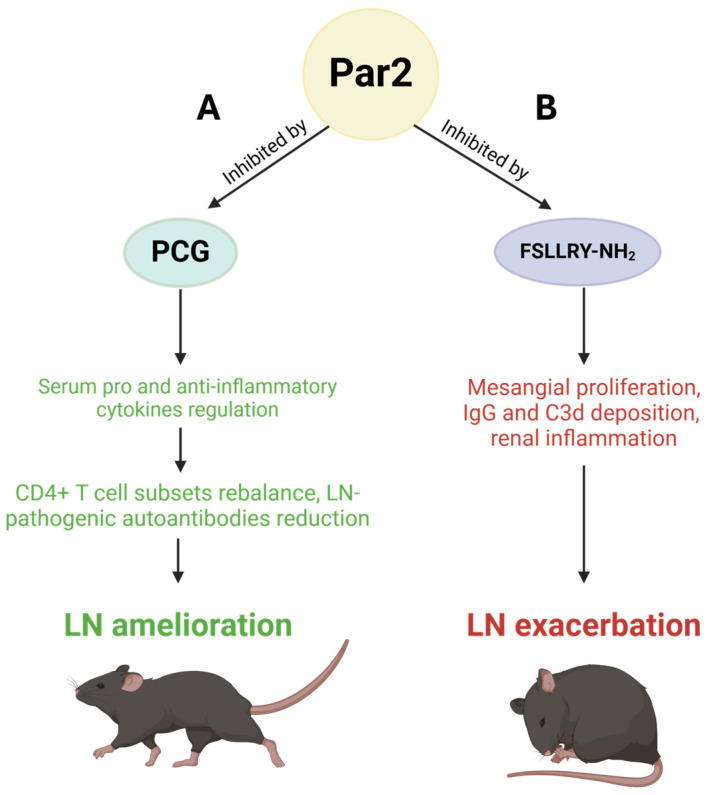
**Par2’s Role in systemic lupus erythematosus (SLE) and lupus nephritis (LN).** (**A**) Par2 inhibition by the antagonist PCG leads to the regulation of pro- and anti-inflammatory cytokine levels, resulting in the rebalancing of CD4+ T cell subsets and a reduction in LN-pathogenic autoantibodies. These effects collectively contribute to the amelioration of LN. (**B**) Conversely, inhibition of Par2 by FSLLRY-NH_2_ induces mesangial proliferation, IgG and C3d deposition, and renal inflammation, which collectively result in the exacerbation of LN.

Newly developed immunosuppressive treatments should aim to shift autoreactive immune cells and their signaling pathways toward an anti-inflammatory state [66]. Punicalagin (PCG), a newly identified Par2 antagonist, could be a promising treatment modality for LN. PCG was found to ameliorate nephritis in lupus-prone mice by the regulation of serum pro- and anti-inflammatory cytokines, subsequently rebalancing CD4+ T cell subsets and reducing LN-pathogenic autoantibodies (Figure 7A) [66].

Itto et al. investigated the effect of a Par2 antagonist, FSLLRY-NH_2_, on the kidney function of lupus-prone MRL/Ipr mice [67]. They aimed to determine the role of Par2 in LN pathogenesis. While they were expecting alleviative effects following the inhibition of Par2, the administration of FSLLRY-NH_2_ aggravated glomerular injury scores, characterized by mesangial proliferation, deposition of IgG and C3d, and renal inflammation, while plasma levels of anti-dsDNA antibody remained unchanged (Figure 7B). This suggests that Par2 activation, in this case, shows protective effects in an SLE murine model [67]. While these findings seem to be contradictory, they correspond to the findings we recently highlighted [8]. A study by Seo et al. [65] showed that Par2 inhibition reduced immune activity, confirming that Par2 inhibition in the immune system reduced immune response. The study by Itto et al. [67] reinforces our recent findings that Par2 activation facilitates regenerative responses. Therefore, in this case, when Par2 was inhibited, it prevented renal regeneration, causing the aggravation of the LN phenotype.

### 2.7. Multiple Sclerosis

Multiple sclerosis (MS) is a chronic, inflammatory, neurodegenerative autoimmune disease of the central nervous system (CNS), noted for the destruction of the myelin sheath structure enveloping the nerves in the brain and spinal cord [68]. This occurs when there is an infiltration of myelin sheath-targeted peripheral immune cells. This loss of myelin results in neuronal damage to the axons and cell bodies [69], leading to adverse cognitive dysfunctions, such as visual, auditory, and sensory delays. In some patients, the complications can be more severe and might lead to movement disorders, seizures, and dementia [70].

The most noteworthy pathophysiological hallmark of MS is blood–brain barrier (BBB) disruption and transendothelial trafficking of immune cells into the CNS. This demyelinating and damage-inducing process is primarily caused by the formation of inflammatory lesions in the brain. Although this process is regulated by the BBB, which is made up of close intracellular tight junctions, its structural integrity might become compromised in inflammatory conditions [71].

**Figure 8:**
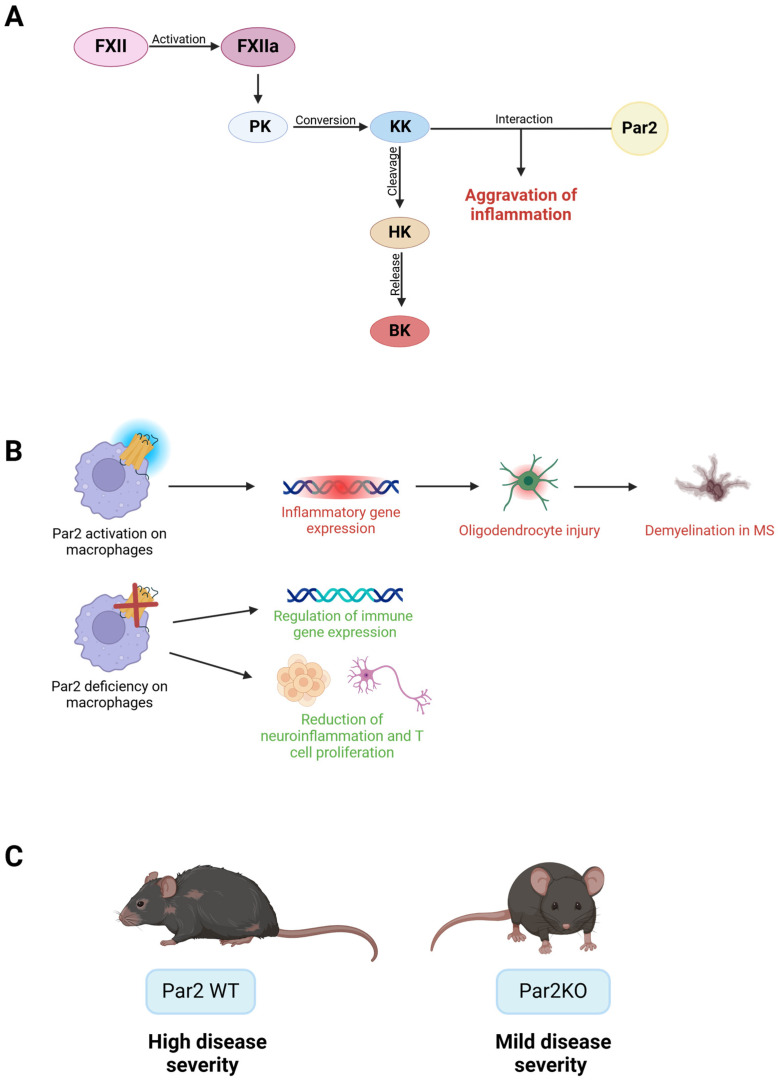
**Role of Par2 in multiple sclerosis (MS) and neuroinflammation.** (**A**) Activation of the kallikrein–kinin system (KKS) contributes to the aggravation of neuroinflammation in MS. Factor XII (FXII) is converted to its activated form (FXIIa), which subsequently converts plasma prekallikrein (PK) to plasma kallikrein (KK). KK then cleaves high-molecular weight kininogen (HK), releasing bradykinin (BK), a proinflammatory peptide. KK can also interact with Par2, thereby further promoting inflammation. (**B**) Par2 singling on macrophages directly contributes to oligodendrocyte injury and inflammatory gene expression, which in turn leads to demyelination in MS. On the other hand, Par2 deficiency on macrophages has reparative effects, regulating the gene expression in immune cells and reducing neuroinflammation and T cell proliferation. (**C**) When Par2 was present (WT mice), the severity of the disease was higher, whereas when it was absent (Par2KO mice), disease severity was relatively mild.

Recent research indicates that components of the coagulation system and the kallikrein–kinin system (KKS) may be involved in MS, suggesting that the pathology is more complex than previously thought. KKS’s plasma kallikrein (KK), derived from plasma prekallikrein, can interact with Par1 AND Par2, further aggravating inflammation and potentially contributing to MS pathology (Figure 8A). Indeed, following this hypothesis, it was shown that Par2 was expressed in isolated mouse brain microvascular endothelial cells (MBMECs) and was upregulated under inflammatory conditions. Additionally, KK influenced migration only when Par2 was present on MBMECs, overall indicating that KK was the key regulator of brain endothelial cells in neuroinflammation, thus making KK a promising target in influencing the BBB and affecting the disease progression in MS [71].

### 2.8. Inflammatory Bowel Disease

Inflammatory bowel disease (IBD) is an inflammatory, chronic condition of the gastrointestinal tract [72]. This immune-mediated disease encompasses both Crohn’s disease (CD) and ulcerative colitis (UC), which share similar symptoms leading to digestive disorders and inflammation within the digestive system [73]. Like other autoimmune diseases, its etiology remains a mystery. Nevertheless, it is thought that genetic predisposition, environmental factors, a dysregulated immune response, and an imbalanced gut microbiome can all contribute to IBD onset, leading to a disrupted intestinal mucosa [72].

CD is an idiopathic, chronic inflammatory disease that can affect any part of the gastrointestinal tract, but the most frequently affected part is the distal ileum [74,75]. CD is characterized by periods of flares and remissions throughout the disease which stem from the disruption of the intestinal mucosa. The development of the disease hinges on tissue inflammation triggered by an unregulated immune reaction to luminal bacterial antigens [75].

UC is a chronic inflammatory condition characterized by episodes of flare-ups and remissions of mucosal inflammation, beginning in the rectum and spreading to proximal sections of the colon [76]. In UC, damage to the mucinous epithelial barrier, which serves as the first line of defense of the mucosal immune system, makes it hyperpermeable. This loss of barrier function allows for greater absorption of luminal antigens, but it remains uncertain whether this dysfunction occurs before ulcerative colitis or is a consequence of ongoing inflammation [77].

**Figure 9:**
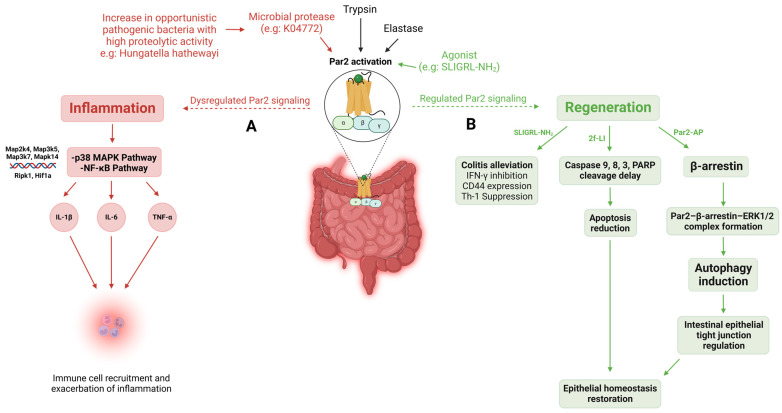
**Par2’s role in inflammation and regeneration in inflammatory bowel disease (IBD).** Par2 can be activated by various proteases: trypsin, elastase, agonists, and microbial proteases. (**A**) Dysregulated Par2 signaling, induced by microbial proteases (e.g., K04772 from pathogenic bacteria like *Hungatella Hathewayi*), activates intracellular signaling cascades involving the p38 MAPK and NF-κB pathways along with their associated genes *Map2k4, Map3k5, Map3k7, Mapk14, Ripk1,* and *Hif1a*, respectively. These pathways then trigger the increased expression of pro-inflammatory cytokines (IL-1β, IL-6, TNF-α), thus contributing to the chronic inflammatory response seen in IBD. (**B**) On the other hand, regeneration takes place upon the regulated activation of Par2. By using the Par2 agonist SLIGRL-NH_2_, colitis alleviation is achieved and indicated by inhibition of Interferon γ (IFN-γ), expression of cluster of differentiation 44 (CD44), and suppression of T-helper 1 (Th-1). Par2 activation with the agonist 2f-LI can lead to the delay of the cleavage of caspase-9, -8, and -3 and PARP, reducing apoptosis and resulting in the restoration of epithelial homeostasis. Alternatively, this restoration can also be achieved using the Par2-activating peptide (Par2-AP), which initiates β-arrestin, forming the Par2–β-arrestin–ERK1/2 complex, inducing autophagy. This helps regulate the intestinal epithelial tight junction and facilitate the restoration of epithelial homeostasis.

Recent studies have highlighted the critical role of Par2 in mediating inflammation. N-terminal-cleaving serine proteases, such as trypsin, elastase, and microbial proteases, can all activate Par2. An imbalance in these proteolytic activities, such as an increase in opportunistic pathogenic bacteria with high proteolytic capacity, can lead to dysregulated Par2 signaling [27]. An example of such bacteria is *Hungatella Hathewayi*, which is a member of the gut microbiome, with the ability to express proteases such as K04772 that specifically cleave Par2, leading to its activation [28]. Upon its activation, the receptor initiates intracellular signaling cascades and subsequently activates the pro-inflammatory p38 MAPK and NF-κB pathways [28,78,79]. Through these pathways, activated Par2 promotes the expression and release of key pro-inflammatory cytokines such as IL-1β, IL-6, and TNF-α. These cytokines play crucial roles in amplifying inflammatory responses and recruiting immune cells to the site of inflammation (Figure 9A).

Santiago et al. [28] demonstrated this by taking germ-free mice and colonizing them with fecal material from CD patients exhibiting high proteolytic activity. These mice developed a pro-inflammatory immune tone, indicating that the proteolytic activity of the microbiota influenced the immune response through Par2 signaling pathways. This proves that Par2 serves as a critical mediator of inflammation in IBD, integrating microbial signals with host immune responses and thereby contributing to the disease pathology observed in conditions like CD and UD [28].

Rondeau et al. revealed that Par2 has a dual role in mediating inflammation by integrating signals from both the host and the microbiota. They showed that the cleavage of Par2 by microbial proteases exacerbated inflammation, contributing to a cycle of increasing intestinal damage [27]. Moreover, they demonstrated the importance of Par2 activation, which leads to the assembly of a signaling module involving MAPK pathways in mediating various cellular responses, including inflammation. Specific genes related to MAPK (*Map2k4, Map3k5, Map3k7,* and *Mapk14*) were upregulated in WT mice but not in Par2KO mice, indicating that Par2 signaling is vital for these inflammatory responses [27]. The NF-κB pathway, which is also stimulated by Par2 activation, leads to the transcription of various pro-inflammatory genes. Key genes associated with this pathway, such as *Ripk1* and *Hif1a*, showed altered expression levels in WT mice compared to Par2KO mice after colitis induction, further implicating Par2 in the inflammatory process [27].

On a restorative note, Par2 also plays a crucial regenerative role in the context of colitis, as it is abundantly expressed in the gastrointestinal tract, as well as intestinal and colonic epithelial cells [80,81,82]. It has been demonstrated that Par2 activation prevents the onset and promotes the healing of T helper cell type 1-mediated experimental colitis in mice, induced by the intrarectal administration of 2,4,6-trinitrobenzene sulfonic acid (TNBS) [83]. With the use of SLIGRL-NH_2_, a Par2 agonist, the role of Par2 in protection against colon inflammation was explored. It was observed that there was a dose-dependent reduction in TNBS-induced colitis with the administration of the agonist compared to the ineffective scramble control peptide, LSIGRL-NH_2_. Unlike the scrambled control, the agonist directly inhibited IFN-γ secretion and CD44 expression on lamina propria T lymphocytes (LPT) (Figure 9B). In vitro testing revealed that Par2 activation directly suppressed Th-1 cytokine production from LP CD4+ T lymphocytes [83].

One of the key features of IBD pathophysiology is the loss of epithelial homeostasis in the gut [84]. One way to compromise this homeostasis is through apoptosis induced by the cytokines IFN-γ and TNF-α, which is mentioned in a study where they described a new role for Par2 in decreasing the kinetics of cytokine-induced apoptosis in colonic epithelial cells [85]. They used selective agonists for Par2, 2f-LI, and SLIGKV, along with trypsin, and discovered that they managed to delay the cleavage of caspase-9, -8, and -3 and PARP when HT-29 cells were exposed to IFN-γ and TNF-α. Par2 was identified as directly responsible, as its knockdown abolished the survival response induced by the Par2 agonist and heightened the sensitivity of HT-29 cells to cytokine-induced apoptosis (Figure 9B).

Regulation of intestinal epithelial permeability is central in IBD, and its alteration can predict the course of the disease [86]. Par2 was found as a key regulator of intestinal epithelial permeability through the process of autophagy induction [87]. This was further investigated in a study where the authors sought to elucidate the association between Par2-regulated autophagy and tight junction (TJ) regulation [88]. They discovered that Par2 regulates intestinal epithelial TJs by inducing autophagy, contributing to the maintenance of intestinal homeostasis by mucosal barrier permeability regulation. This Par2-mediated autophagy pathway relies on β-arrestin signaling, which is induced by Par2-activating peptide (Par2-AP), leading to the formation of the Par2–β-arrestin–ERK1/2 complex (Figure 9B). This pathway is especially active under inflammatory conditions and helps stabilize TJ integrity by enhancing autophagy [89]. Experiments with autophagy inhibitors, such as chloroquine (CQ), confirmed that autophagy inhibition decreased TJ protein levels and weakened epithelial resistance, highlighting the importance of autophagy in barrier function. Activation of Par2 seemed to alleviate CQ-induced inhibition of autophagy. Overall, Par2’s role in inducing autophagy is key to preserving intestinal barrier integrity, making it a potential therapeutic target for conditions like leaky gut and inflammation [88].

## 3. Discussion

Protease-activated receptor 2 (Par2) has emerged as a multifaceted modulator of immune and tissue responses in autoimmune diseases. Our review highlights the paradoxical roles of Par2, ranging from exacerbating inflammation to promoting tissue healing. This duality underscores the complexity of Par2 signaling and its potential as both a therapeutic target and a challenge in treatment strategies.

In autoimmune conditions such as autoimmune hepatitis, type 1 diabetes, rheumatoid arthritis, Sjögren’s disease, systemic lupus erythematosus, multiple sclerosis, and inflammatory bowel disease, Par2-mediated inflammation has been linked to the amplification of inflammatory cascades through pathways like NF-κB and MAPK. These pathways drive the expression of pro-inflammatory cytokines and mediators, contributing to disease progression. However, Par2’s activation also facilitates repair mechanisms in damaged tissues, such as epithelial barrier restoration in IBD and β-cell survival in T1D. While not all experimental models presented here provide examples where tissue regeneration was induced by Par2 activation, we suggest that revisiting these models with the appropriate experimental approaches could test this hypothesis. Our findings suggest that the context and location of Par2 activation, whether in immune cells or damaged tissues, determine its effects, which can range from destructive inflammation to regenerative healing. In addition, the works presented for IBD and lupus indicate that elucidating the molecular mechanisms governing the decision between inflammatory aggravation and healing is possible [27,66,67,78,79,83,85,88]. Therefore, future studies in autoimmune hepatitis, T1D, RA, and Sjögren’s disease may shed more light on the molecular decision-making mechanisms and determine if the pathways described for IBD and lupus are shared in other autoimmune diseases as well.

The insights gained from our laboratory reconcile some of the previously conflicting data regarding Par2’s role in inflammation and regeneration. By distinguishing between its immune-mediated and tissue-specific effects, we have advanced our understanding of how Par2 influences the course of autoimmune diseases. For example, our studies demonstrated that Par2 activation in immune cells worsens inflammation, while activation in non-immune tissues enhances healing and protects against further damage [8,29,30].

Protease-activated receptor 2 (Par2) presents a promising therapeutic target due to its dual functions in inflammatory and reparative pathways. Par2 antagonists have been shown to suppress excessive inflammation in immune-mediated conditions by inhibiting pro-inflammatory signaling cascades, such as NF-κB and MAPK. Conversely, in injured tissues, selective activation of Par2 could enhance healing by promoting cellular regeneration and maintaining epithelial integrity. These findings underscore the potential for context-specific therapies targeting Par2. However, significant challenges remain, including the development of tissue-specific modulators and the mitigation of off-target effects. Advances in delivery mechanisms, such as nanoparticle-based targeting, may provide viable solutions to these challenges.

These observations hold significant implications for therapeutic development. The ability to selectively modulate Par2 signaling offers a promising avenue for treating autoimmune diseases. For instance, targeting Par2 in immune cells may suppress pathological inflammation, while enhancing its activity in tissues could promote healing and repair.

## 4. Conclusions

Par2’s dual roles in inflammation and tissue healing underscore its potential as both a therapeutic target and a challenge in autoimmune disease research. Advances in understanding its context-specific functions have illuminated new pathways for intervention, offering the potential to harness its reparative effects while mitigating its inflammatory contributions.

Despite these advances, significant challenges remain. Translating this knowledge into therapies requires the development of selective Par2 modulators that can differentiate between its pro-inflammatory and reparative roles. Further research must focus on identifying context-specific molecular targets that can modulate Par2 activity with minimal off-target effects. Moreover, investigating its interplay with other signaling pathways could yield synergistic approaches to mitigate inflammation while enhancing tissue repair. Such work is crucial for transforming Par2’s duality from a challenge into an opportunity for autoimmune disease management.

Future therapeutic strategies must prioritize precision, focusing on selectively modulating Par2 activity within specific cellular and tissue contexts. Such approaches hold the promise of not only addressing the symptoms of autoimmune diseases but also promoting long-term healing. As research into Par2 progresses, it may provide a transformative avenue for personalized treatments, ultimately improving the lives of patients with autoimmune conditions.
